# Health labour market policies in support of universal health coverage: a comprehensive analysis in four African countries

**DOI:** 10.1186/1478-4491-12-55

**Published:** 2014-09-26

**Authors:** Angelica Sousa, Richard M Scheffler, Grayson Koyi, Symplice Ngah Ngah, Ayat Abu-Agla, Harrison M M’kiambati, Jennifer Nyoni

**Affiliations:** Human Resources for Health, Department of Health Systems Policies and Workforce, World Health Organization, Geneva, Switzerland; School of Public Health and the Goldman School of Public Policy, University of California, 50 University Hall, MC 7360, Berkeley, CA 94720 USA; Institute of Economic and Social Research (INESOR), University of Zambia, Lusaka, Zambia; Subregional Institute of Statistics and Applied Economy (ISSEA), Yaoundé, Cameroon; National Human Resources for Health Observatory, Ministry of Health, Khartoum, Sudan; Technical Planning and Coordination, Ministry of Medical Services, Nairobi, Kenya; Human Resources for Health, Division of Health Systems and Services, World Health Organization Regional Office for Africa, Brazzaville, Congo

**Keywords:** Africa, Cameroon, Health labour market, Health workforce, Health workforce policies, Human resources for health, Kenya, Sudan, Universal health coverage, Zambia

## Abstract

**Background:**

Progress toward universal health coverage in many low- and middle-income countries is hindered by the lack of an adequate health workforce that can deliver quality services accessible to the entire population.

**Methods:**

We used a health labour market framework to investigate the key indicators of the dynamics of the health labour market in Cameroon, Kenya, Sudan, and Zambia, and identified the main policies implemented in these countries in the past ten years to address shortages and maldistribution of health workers.

**Results:**

Despite increased availability of health workers in the four countries, major shortages and maldistribution persist. Several factors aggravate these problems, including migration, an aging workforce, and imbalances in skill mix composition.

**Conclusions:**

In this paper, we provide new evidence to inform decision-making for health workforce planning and analysis in low- and middle-income countries. Partial health workforce policies are not sufficient to address these issues. It is crucial to perform a comprehensive analysis in order to understand the dynamics of the health labour market and develop effective polices to address health workforce shortages and maldistribution as part of efforts to attain universal health coverage.

## Background

Universal health coverage is defined as access of all people to comprehensive health services (including prevention, promotion, treatment, and rehabilitation) at affordable cost and without financial hardship, through protection against catastrophic health expenditures [1]. However, even when financial protection is ensured, access to health services will not be guaranteed without an adequate health workforce. Health workers are a fundamental and instrumental component of service delivery. Unless countries have the appropriate health workforce to deliver quality services, universal health coverage will not be attained. This is a particular challenge for many low- and middle-income countries, which often suffer from severe shortages and maldistribution of health workers [2–4].

These problems, however, are not exclusive to low- and middle-income countries; many developed countries are likely to face severe shortages and maldistribution of health workers as a consequence of the latest global economic downturn of 2008/2009. Many wealthy countries are cutting their budgets for social services, including health, which will affect the number of health workers trained and deployed^a^. In addition, the socioeconomic consequences of the financial crisis – increased unemployment rates, poverty, and social deprivation – coupled with emerging issues, such as aging populations and a higher prevalence of chronic conditions, will increase the demand for public health services in both developed and developing countries [5]. Moreover, the new dynamics of migration – for example, moving from one developing country to another – pose a major challenge for global health labour markets [6]. To cope with these challenges and attain universal health coverage, countries will have to do in-depth analyses of their health labour markets in order to understand the driving forces that affect supply and demand both within the country and at a global level [5].

In this paper, we use a health labour market framework to investigate the key indicators of the dynamics of the health labour market in Cameroon, Kenya, Sudan^b^, and Zambia, and identify the main policies implemented in the past decade to address shortages and maldistribution of health workers in order to highlight the challenges to attaining universal health coverage. These four African countries were chosen because they had reliable and good quality data and the necessary institutional capacity to conduct the analysis, and the Ministry of Health was in each case willing to undertake the analysis^c^.

The four countries differ in their overall level of economic development and their demographic and epidemiological profiles (Table 1). In each of the countries, there are great inequalities in access to health services and difficulties in attaining universal health coverage. For example, in Cameroon, Kenya, and Zambia, the coverage of skilled birth attendance is less than 20% among the poorest segments of the population, compared with more than 80% for the rich [7] (Table 2).

**Table 1:** **Sociodemographic indicators for the four countries** ^**a**^ **in 2011**

Indicators	Cameroon	Kenya	Sudan	Zambia
**Population**	20,386,799 [8]	41,609,700	30,894,000 [9]	13,046,508 [10]
**GDP per capita in PPP terms** [11]	1,151	865	1,580	1,469
**Expenditure on health (% of GDP)** [7]	5.2	4.5	8.4	6.1
**Under-five mortality rate (per 1000 live births)** [7]	74	108	89	50
**HIV prevalence (%)** [7]	4.6	6.2	0.4	12.5
**Main causes of death**	Malaria, HIV/AIDS	HIV/AIDS, malaria, and tuberculosis	Heart diseases, septicemia, respiratory infections, malaria, diarrheal diseases, gastroenteritis [12]	HIV/AIDS, tuberculosis, and malaria [13]

Sources: ref. [14].

GDP, Gross domestic product; PPP, Purchasing power parity; HIV, Human immunodeficiency virus; AIDS, Acquired immunodeficiency syndrome.

^a^Data are for 2011 or the most recent year available. Countries are given in alphabetical order. Numbers in square brackets give the reference number of the source of the data.

**Table 2:** **Sociodemographic indicators available in the four countries** ^**a**^ **in 2002**

Indicators	Cameroon [15]	Kenya [16]	Sudan [12]	Zambia [13]
**Population**	16,018,000	31,987,000	33,610,000	10,812,000
**GDP per capita in PPP terms**	2,037	1,020	1,806	839
**Expenditure on health (% of GDP)**	4.6	4.9	4.9	5.8
**Under-five mortality rate (per 1,000 live births)**	164	126	95	191

Sources: ref. [11–13, 15, 16].

GDP, Gross domestic product; PPP, Purchasing power parity; HIV, Human immunodeficiency virus; AIDS, Acquired immunodeficiency syndrome.

^a^Data are for 2002. Countries are given in alphabetical order. Numbers in square brackets give the reference number of the source of the data.

### The health labour market framework

In many low- and middle- income countries, health workforce policies are frequently formulated on the basis of the number of health workers required to meet the needs of the population (needs-based assessment), and are aimed at training more health workers. However, unless appropriate employment conditions are in place to absorb the newly trained health workers into the labour market, there is a risk of increasing unemployment and brain drain, and wasting resource [17].

The level of employment in a country’s health sector is in large part determined by health labour market dynamics, not the population health needs or the worker education capacity alone. The health labour market encompasses a number of dimensions. The combination of needs, demand, supply, training, and governance of health workers determines the wages and allowances, the number of health workers employed, the number of hours they work, their geographic location, their employment setting, their productivity, and their performance [18].

The education and training of health workers is a key determinant of the supply of health labour in a country. The availability of new graduates depends on a number of factors, such as the number of slots available in training programs and the admissions criteria, the location and social orientation of the medical training, and the debt burden. It also depends on individual decisions by potential health workers whether to pursue a health profession and obtain the required education. Those decisions will be based, at least in part, on the attractiveness of the salaries and potential returns for the years of study and financial investment needed (e.g., for tuition fees, books, etc.). The pool of qualified health workers is the number of individuals who have been trained, but the supply is only the number of qualified health workers willing to work at the prevailing wage rate.

The demand for health care workers is determined by the private and public institutions that hire and pay health workers in clinics, hospitals, and other settings. These institutions will compete for workers based on their wage rates, budgets, provider payment practices, benefits packages, working conditions, and other labour regulations and rules. The competitiveness of institutions across all of these factors will influence how attractive they are to health professionals (including new graduates) in comparison with other labour markets or other countries [19]. Opportunities for self-employment also affect the demand for health workers.

In general, the higher the wage, the larger the number of available health workers willing to work in the health sector. However, the wage is not the only determining factor. Good working conditions, safety, availability of supplies and technical support, and career opportunities also play important roles. Taken together, these factors influence whether health professionals decide to work in the country (or migrate), in the health sector (or in another sector), in a public institution (or a private one), on a full-time basis (or part-time), and in an urban area (or a rural one).

In many countries, health worker shortages occur because of labour market mismatches rather than because of an overall lack of workers to fill the jobs in question.

### Methods and data

To understand the health labour market dynamics in the four selected countries, we investigated the factors that determine the health workforce supply and demand. Fourteen key indicators were selected for cross-country comparisons. These were grouped into two categories: stock and density indicators, and shortage indicators (Table 3). The first group, stock and density indicators, includes measures of the available health workforce, their age, sex, skill mix, geographical location, distribution by sector, presence in the informal economy, and migration. The available health workforce is defined as anyone with the training and ability to do the job, whether they are employed in that position or not. The second group, shortage indicators, reflects the shortages of health workers from different perspectives:

**Table 3:** **Selected health labour market indicators in the four countries** ^**a**^ **in 2011**

	Cameroon ^h^	Kenya	Sudan	Zambia
**Stock and density indicators 2011**				
**Density of health workers (per 1,000 population)**	1.87	0.8	2.36	1.41
	(total no. 38,207)	(total no. 31,060)	(total no. 77,280)	(total no. 18,397)
**Density of physicians (per 1,000 population)** ^**b**^	0.1	0.1	0.6	0.2
	(total no. 1,842)	(total no. 4,660)	(total no. 19,778)	(total 2,419)
**Density of nurses and midwives (per 1,000 population)** ^**c**^	0.93	0.45	1.02	0.86
	(total no. 18,954)	(total no. 18,749)	(total no. 33,193)	(total no. 11,193)
**Distribution of health workers by category**	4.8% physicians, 49.6% nurses, 45.6% others	15% physicians, 60.4% nurses and midwives, 24.6% others	25.6% physicians, 43% nurses and midwives, 31.4% others	13.2% physicians, 60.8% nurses and midwives, 26% others
**Percentage over 40 years old**	43 (nurses) 46 (physicians)	70 (nurses)	49 (all health workers)	56 (midwives)^d^
**Percentage female (all health workers**	56	60	52	61
**Percentage of health workers employed in public sector** ^**e**^	66	33	62	79
**Categories of health workers in informal economy**	0.5% nurses, support staff and paramedical staff^f^	traditional medicine practitioners, traditional birth attendants	community health workers, village midwives	traditional birth attendants, traditional attendants
**Migration**	Data not available	51% physicians, 8.3% nurses [20]	60% physicians, 25% pharmacists	0.5% of nurses
**Main destination countries**	Data not available	USA, Australia, Namibia	Saudi Arabia, United Kingdom, Ireland	United Kingdom, Swaziland, Botswana
**Geographical distribution (per 1,000 population)**	Highest density 3.2 (Yaoundé) lowest density 0.7 (North province)	Highest density 1.81 (Central province) lowest density 0.84 (North Eastern province)	Highest density 3.2 (Northern state) lowest density 0.7 (South Darfur)	Highest density 1.66 (Lusaka (urban) and Copperbelt) lowest density 0.68 (Northern province (rural))
**Shortage indicators**				
**Needs-based shortage of health workers**	55% overall; 65% physicians, 42% nurses	70% overall; 59% physicians, 57% nurses and midwives	57% physicians, 61% nurses and midwives	54% overall; 63% physicians, 50% nurses and midwives
**Economic shortage of health workers (vacancy rate)**	Data not available	39% physicians, 33% nurses and midwives	25% overall; 36% physicians, 21% nurses^g^	62% physicians, 53% nurses and midwives
**Wage shortage** ^**h**^	Physicians’ wage 6.8 times average income per capita; nurses’ wage 3.8 times average income per capita	Physicians’ wage 44 times average income per capita; nurses’ wage 15 times average income per capita	Physicians’ wage 55% more than average income per capita; nurses’ wage same as average income per capita	Physicians’ wage 15 times average income per capita; nurses’ wage 7.5 times average income per capita

Sources: refs [20–23].

^a^Data are for 2011 or the most recent year available. Countries are given in alphabetical order. Numbers in square brackets give the reference number of the source of the data.

^b^The term *physician* includes following health worker categories: doctors and medical assistants for Sudan; physicians, medical licentiates, and clinical officers for Zambia; medical officers for Cameroon; medical officers and registered clinical officers for Kenya.

^c^The term *nurses and midwives* includes following health worker categories: midwives and nurses for Sudan and Zambia; nurses for Cameroon (no cadre of midwives is listed in the available data); bachelor of science in nursing and registered community health nurses for Kenya. In Cameroon and Kenya, midwives are not listed as a separate cadre.

^d^This number refers to midwives older than 45 years.

^e^Private includes both profit and non-profit.

^f^This number is likely to be an underestimate, as most informal workers were not included in the census.

^g^Calculated from a health facility survey conducted in 2011 in six states: Blue Nile, Kassala, Khartoum, Northern Kordofan, Red Sea, Southern Kordofan.

^h^Not adjusted for allowances and converted to US$ using the current exchange rate (Kenya: US$1 = 85.77 KES; Sudan: US$1 = 2.5 SDG.

1. The economic shortage, defined by the vacancy rate (the ratio of unfilled vacancies to funded health care posts), to identify the gap between demand and supply;
2. The wage shortage (the ratio of the average health worker wage to the country’s per capita GDP) to identify the gap between the wages in the health sector and the country as a whole; and
3. The needs-based shortage (the gap between the available health workforce and the workforce required to meet the health needs of the population), to identify health sector deficiencies in meeting population needs.

The main sources of data were official statistics from the Ministry of Health, the Ministry of Finance, health professional councils, population censuses, and labour force surveys [20–23]. We additionally used the framework and policy levers proposed by Sousa et al. [5] to identify the major policies that have been implemented in the past ten years in these four countries to address different dimensions of the health labour market.

## Results

### Health labour market indicators

The available supply of health workers varies from country to country (Table 3). The wealthiest of the four countries, Sudan, has the highest density of total health workers and physicians per 1,000 population, while Kenya and Cameroon, the poorest countries, have the lowest density. In the past ten years, there have been notable increases in the availability of health workers in these four countries, with an average annual growth rate of around 5%.

Although all four countries increased in population over the past decade, with Kenya increasing the most by 33% of its 2004 population, the overall density of health workers decreased in all four countries according to the World Health Statistics. Sudan experienced the smallest health workforce density decrease of 0.19 whereas Zambia’s decreased the greatest by 2.39 (Table 4). As for physician density, Sudan’s density significantly surged while Kenya’s grew only slightly. On the contrary, Cameroon’s physician population decreased by half from 200 to 100 per 1,000 from 2004 to 2011. Zambia also experienced a decrease in physician density but less extreme than Cameroon. Sudan and Zambia both showed contractions in the density of health workers, while the density of physicians and nurses escalated revealing that other health worker positions decreased and that these specific occupation populations surged. Over this period of time, the percent of GDP per country remained relatively stagnant, except in the case of Sudan, which rose 3.5% bringing it to 8.4%.

**Table 4:** **Selected health labour market indicators in the four countries** ^**a**^ **in 2004 and difference in indicators from 2004 to 2011**

	Cameroon [15]	Kenya [16]	Sudan [12]	Zambia [13]
**Stock and density indicators 2004**				
**Density of health workers (per 1,000 population)**	2.34 (total no. 37,752)	1.2 (total no. 38,922)	2.55 (total no. 99,538)	3.8 (total no. 41,429)
**Density of physicians (per 1,000 population)** ^**b**^	0.192 (total no. 3,124)	0.037 (total no. 1,203)	0.22 (total no. 7,552)	0.137 (total no. 1,499)
**Density of nurses and midwives (per 1,000 population)** ^**c**^	1.598 (total no. 26,042)	0.498 (total no. 16,146)	0.917 (total no. 31,496)	2.015 (total no. 22,010)
**Distribution of health workers by category**	8.2% physicians, 69% nurses and midwives, 22.8% others	3.1% physicians (significantly less than 2002 or 2008), 41.5% nurses and midwives, 55.4% others	7.6% physicians, 31.6% nurses and midwives, 60.8% others	3.6% physicians, 53.1% nurses and midwives, 43.3% others
**Difference in indicators from 2004 to 2011**				
**Density of health workers**	−0.47	−0.4	−0.19	−2.66
**Density of physician**	−0.09	−0.06	0.38	0.06
**Density of nurses and midwives**	−0.668	−0.05	0.1	1.16

Sources: refs [11–13, 15, 16].

^a^Data are for 2004 or the most recent year available. Countries are given in alphabetical order. Numbers in square brackets give the reference number of the source of the data.

^b^The term *physician* includes following health worker categories: doctors and medical assistants for Sudan; physicians, medical licentiates and clinical officers for Zambia; medical officers for Cameroon; medical officers and registered clinical officers for Kenya.

^c^The term *nurses and midwives* includes following health worker categories: midwives and nurses for Sudan and Zambia; nurses for Cameroon (no cadre of midwives is listed in the available data); bachelor of science in nursing and registered community health nurses for Kenya. In Cameroon and Kenya, midwives are not listed as a separate cadre.

The health workforces in Kenya and Zambia are mainly composed of nurses and midwives, accounting for 60% and 61% of the total, respectively. However, a large proportion of nurses in Kenya and of midwives in Zambia are over 45 years old, suggesting that unless strategies are put in place to recruit younger staff, these countries will soon experience a shortage of health workers. In Cameroon, there is a mix of different cadres of health workers with different specialties and training levels; strategies for task shifting have been effective in allowing workers other than physicians and nurses to deliver health services (see Table 5 for a summary of the main policies).

**Table 5:** **Summary of the main health labour market policies in four countries, 2000–2011** ^**f**^

Type of policy	Cameroon		Kenya		Sudan		Zambia	
	Policies	Effect	Policies	Effect	Policies	Effect	Policies	Effect
**Policies to increase the production of health workers**	1. Investment in health training institutions since 2000.	Positive effect: this policy together with policy to grant subsidies to private sector increased the number of trained medico-sanitary workers from 2,285 in 2000 to 3,307 in 2011 (69%).	1. National health training policy in 2009.	Negative effect: lack of coordination among training agencies resulted in inefficiencies, duplication of effort and wastage of resources. Lack of investments in training despite increase in demand; thus, the number of graduates in various cadres, e.g., nurses, has steadily decreased.	1. Revolution of Higher Education (1996).	Positive effect: About 10 new universities were opened and intake increased. The number of medical schools increased 8 times between 1996 and 2012. The number of doctors graduating each year increased from 400 to 1,400. Number of health workers in the public sector increased. Negative effect: increase in unemployment among new graduates, e.g., junior doctors.	1. Reopening of closed public training institutions, increased number of scholarships for in-service training programs, provision of financial retention incentives for teaching staff (within past five years).	Positive: number of graduates increased from 1,101 in 2007 to 2,311 in 2010 (increase of 110%).
	2. Reform of the Faculty of Medicine and Biomedical Sciences (FMBS) with the opening of new training branches; creation of 3 new faculties of medicine (2007).	Positive effect: increased number of students in the faculty. Education of a medical officer requires at least 7 years, thus the benefit in terms of more health workers will be effective from 2014.	2. Facilitate rational development of the health workforce through alignment of curricula and training needs, create pre-service scholarships, establish cadre-based colleges, and establish medical training colleges in every county by 2017.	Positive effect: increased enrolment of students in the areas of need. Negative effect: the transition phase as devolution takes effect will bring some confusion in the health system.	2. Sudan Declaration for Allied Health Professionals (2004) to scale up the education of nurses, midwives, and allied health professions.	Positive effect: increased intake in different universities; a bridging programme was adopted to raise educational level of vocationally-trained cadres. Negative effect: universities could not expand the intake of nurses, midwives, and allied health professions to meet the needs, which resulted in the initiation of the Academy of Health Sciences.	2. Training community health workers to improve access of rural population to health services, 2010.	Positive effect: 307 community health workers graduated in 2011 and all have been placed in health posts in rural areas; 290 were enrolled for training in 2012.
			3. Institutionalization of competence- based training programs, e.g., e-learning programme for nurses.	Positive effect: increased number of nurses trained in rural areas.	3. Decentralized Health Professional Education (HPE) through Academies of Health Science (in 2005) to ensure that programs are established according to the needs of the state and increase the training capacity of the allied health academy.	Positive effect: increased production of nurses, midwives, and allied health professions and improved skill mix composition of health workers; 6,000 new graduates from nursing, midwifery, and allied health professions recruited within their states.		
**Policies to address inflows and outflows**	1. Performance-based financing approach in Eastern region since 2006. Now extended to other regions (North West, South West, and Littoral).	Positive effect: improvement in quality and quantity of care delivered, more resources collected by involved health centres, and better remuneration of staff.	1. Introduction of hardship and commuter allowances; improve data collection on health worker mobility and retention to curb out migration of health workers within and without the country since 2011. Improve working conditions (since 2009).	Positive effect: decreased migration of nurses. Negative effect: has not been enough to stop exits of medical doctors (50% between 2005 and 2009) and enrolled community nurses (81%).	No national policy to address inflows and outflows.	No national policy to address inflows and outflows.	1. Policies to attract foreign health workers with same conditions as nationals, except that foreign nationals are employed on renewable fixed-term three-year contracts.	Positive effect: The percentage of doctors increased by 30%.
			2. Structural adjustment programme (before 2007) resulted in employment freeze of civil servants (with the exception of doctors). Freeze reversed in 2009 with expansion and opening of new facilities and upgrading of existing ones.	Positive effect: a significant reduction in vacancy rates; an increase in staff of 2,793, representing 8.4% growth between 2009 and 2010. Negative effect: the employment freeze resulted in a long-term decline in the number of civil servants including health workers; as a consequence, some cadres, such as nurses, medical laboratory, and dental technologists, have a high proportion of over 45-year-olds.			2. Improve employment conditions for health workers, and introduce in-service training and opportunities for progression within the public sector. More intense implementation since 2003.	Positive effect: decreased migration of nurses, from 99 in 2007 to 44 in 2011.
			3. The retirement age of civil servants raised from 55 to 60 years in 2009.	Effect: changed the age profile of public sector health workers, which may result in high rates of unemployment and brain drain among new graduates, who will have difficulty finding a job in the health sector.			3. Public sector incentives to improve wages. Deliberate policy since 2005 to improve salaries for health workers.	Positive effect: decreased migration of nurses from 99 in 2007 to 44 in 2011. Negative effect: vacancy rates not necessarily decreased because staffing requirements keep changing with growing demand for services.
**Policies to address maldistribution and inefficiencies**	1. Emergency plan for upgrading quantity and quality of workforce (2006–2008).	Positive effect: helped recruit nearly 2,500 health workers in remote areas with severe needs for health workers. Negative effect: donor funding of programme came to an end; health workers have the right to move.	1. Review workforce norms and standards, measure performance of health workforce, establish frameworks to manage and monitor health workers, improve performance standards, strengthen supervision and accountability by 2030.	Positive effect: decreased inequality in the distribution of health workers and improved quality of services. Negative effects: main challenge is lack of functional infrastructure for service delivery, which undermines the performance of health workers.	1. Policy on deployment of medical specialists adopted in 2002. A contract with the Ministry of Health offers specialist training in exchange doctors agreeing to work in states according to health needs.	Negative effect: doctors may not abide by the contract and recipient states may not provide competent training services.	1. Compensation scheme for medical doctors serving in rural areas introduced in 2003 and expanded to other health workers in 2007.	Positive effect: this programme helped increase the number of health workers in rural areas between 2005 and 2010. Limited effect: no reduction in shortages of health workers in remote areas, because of effect of other factors, such as living conditions, safety, infrastructure, and job opportunities.
	2. Programme to encourage employment in difficult areas (supported by C2D funding) launched in 2012.	Too early to measure effects.	2. Emergency hiring programme (2006) to provide a 3-year contract for health workers who work in underserved areas, recruit local health workers and provide hardship allowances, housing grants and paid leave.	Positive effect: increased number of health workers in rural areas. Negative effect: disharmonized remuneration.	2. Policy on deployment of medical specialists adopted in 2002: every medical or health graduate is obliged to work one year as national service; handled by the Ministry of Defence, which distributes them throughout the country to address geographical maldistribution.	Negative effect: the new graduates are young and inexperienced.	2. The UN Population Fund introduced a bonding system, in which nursing students in Northern Western Province received a bursary for payment of tuition fees from 2003.	Positive effect: improved staffing and retention of nurses in the province, as students are bonded for a period of 2 years after graduation.
	3. Enhance staff motivation with payment of “productivity allowance” (10% of financial resources generated by the facility activities) since 1994.	Positive effect: improved quality of care delivered; Negative effect: no proof of reduced out-migration.	3. Develop a national electronic database for nurses to better match nurses to underserved areas in 2005.	Positive effect: reduced unfilled rural posts and faster recruitment.	3. Grant specialization scholarships for doctors practising in hardship areas. Provide incentive packages to retain doctors.	Positive effect: increased rotation of health workers in underserved areas. Negative effect: sporadic efforts not sustained.		
**Policies to regulate public and private sectors**	1. Subsidies granted to private sector since 2000.	Positive effect: (together with investment in health training institutions) number of trained medico-sanitary workers increased from 2,285 in 2000 to 3,307 in 2011 (69%).	1. Launch distance e-learning through public-private partnership, establish interagency coordinating committee for human resources for health (HRH), form 26-member multi-stakeholder group for HRH	Positive effect: improved harmony in addressing HRH issues. Negative effects: difficult to convince some stakeholders to prioritize the issues raised in the health sector.	1. Directorate of Planning of the Federal Ministry of Health in 2010 adopted a policy towards private sector.	Negative effect: The Ministry of Health has weak regulatory and monitoring tools.	1. Encourage private sector participation in pre-service training.	Positive effect: (together with other policies) number of graduates increased from 1,101 in 2007 to 2,311 in 2010 (increase of 110%).

Sources: refs [20–23].

Notes: Countries are given in alphabetical order.

In the four countries, the majority of health workers are women: for example, in Sudan 67% of medical students are women, and in Kenya and Zambia 60% of all health workers are women. This implies that further policies should be oriented to making the working environment friendly for women.

In Cameroon, Sudan, and Zambia, the main employer of health workers is the public sector; however, a large proportion of health workers work in both the public and the private sector. In Sudan, for example, 90% of health workers have a dual practice. However, since there are no policies to regulate it, there are concerns about the availability of health workers in practice and about the quality of services. This suggests that further actions should be oriented to properly regulating and understanding the implications of dual practice employment and its impact on the quality of health services. There is also the need to incorporate informal health workers into the formal economy as in all four countries there is a proportion of the population covered by unregulated health providers such as traditional birth attendants.

In Kenya and Sudan, migration – particularly of physicians – is a major problem. This suggests that there are a number of qualified physicians who would rather work abroad for better working conditions and higher wages than in their home country with the current working conditions and wages.

Despite efforts to decrease health workforce inequalities between poor and non-poor areas (see Table 5 for a summary of the main policies) major inequalities remain in all four countries. In Kenya and Zambia, the best-served areas have approximately twice as many health workers as the areas with the lowest density; in Cameroon, the capital city (Yaoundé) has 4.5 times more health workers per 1,000 population than the poorest province (North), which also has the lowest coverage of skilled birth attendants. These data imply that poorer areas have difficulty attracting and retaining health workers, and therefore the population has less access to health services and worse health outcomes than the population in better-off areas. This lack of health workforce capacity to provide health services to the entire population is a major challenge for ensuring equitable access to health services and universal health coverage. Thus, policies should be directed to retaining health workers in underserved and poor areas.

Despite the efforts made to decrease the shortages of health workers (see Table 5 for a summary of the main policies), such shortages remain a critical constraint for service delivery. The needs-based shortage measure suggests that, in each country, the health sector has less than half of the health workers required to meet the needs of the population.

A higher probability of labour shortage is typically associated with a higher vacancy rate. In Zambia, the particularly high rate of unfilled vacancies (62% of physician posts unfilled and 53% of nurse and midwife posts unfilled) suggests that the number of health workers that employers are willing to hire exceeds the number who is willing to work with the proposed working conditions and wages.

Wages are a key component of labour markets. Some governments need to pay higher wages than at present to keep physicians and nurses they are currently training in the health sector and in the country. Between 1990 and 2004, Zambia experienced an exodus of physicians. To discourage more physicians from leaving the country, the government increased the wages of physicians by 16% between 2007 and 2011, to a level 15 times higher than the average income per capita and higher than that of other professions with a similar level of education, such as lawyers. However, despite this increase, the average annual wage of physicians is still only US $21,780.

These findings suggest that there are problems on the demand and the supply side. Unless policies to address health worker shortages and maldistribution are designed with a health labour market perspective, they are unlikely to be effective.

### Health labour market policies

We used the framework and policy levers proposed by Sousa et al. [5] to identify the major policies that have been implemented in the past ten years in the four countries and their impact on: 1) the production of new graduates; 2) inflows and outflows of health workers; 3) maldistribution of health workers and inefficiencies; and 4) regulation of the private sector (Table 5).

#### Production of new graduates

In the past ten years, major efforts have been undertaken by all four countries to decrease the shortages of health workers. On the production side, the most significant efforts include opening new training institutions, awarding scholarships, providing financial incentives for teaching staff, and training new cadres of health workers. These policies have been highly effective in increasing the number of new graduates in Cameroon and Zambia. However, in Kenya and Sudan, the policies have had limited or negative effects, as they did not take into account the labour market dynamics. In Sudan, for example, there was an increase in unemployment among new graduates, as vacancies and funding were not adjusted to absorb the new graduates into the labour market. The result was a waste of resources and increased brain drain.

#### Inflows and outflows of health workers

Inflows and outflows refer to the movement of health workers into and out of the country as well as into and out of the overall health workforce. Efforts to address inflows and outflows of health workers have included policies to increase wages and provide extra allowances, improve working conditions, and provide training opportunities. In Zambia, for example, policies targeted at increasing wages and allowances^d^ were effective in reducing migration of nurses but not effective in decreasing the vacancy rate – although more nurses remained employed, there was still a large pool of unmet needs that increased over time. In Kenya, the introduction of allowances reduced the migration of nurses, but was not sufficient to reduce exits^e^ of medical doctors and community nurses. Kenya also implemented specific policies to increase the retirement age of civil servants, from 55 to 60 years, to tackle the problem of aging nurses and other cadres of health workers. These efforts have changed the age profile of the health workforce; however, they may result in high rates of unemployment and brain drain among new graduates, who will have difficulty finding a job in the health sector, compromising the availability of health workers in the medium and long term and, therefore, the attainment of universal health coverage.

#### Maldistribution of health workers and inefficiencies

Several policies have been implemented to address maldistribution and inefficiencies of health workers such as training local health workers, providing allowances, and awarding scholarships to increase the supply of health workers in underserved and rural areas. Kenya launched an e-learning programme to train health workers in rural areas, while Zambia trained community health workers to work in underserved areas. These policies have been effective in increasing the number of health workers in rural and underserved areas. However, they have not been sufficient to reduce health worker shortages and increase access to quality services for the entire population. In Zambia, the provision of compensation schemes for health workers serving in rural areas was effective in increasing the number of health workers in the targeted areas. However, it did not lead to a decrease in the overall shortage of health workers. In Sudan, several efforts have been made to increase access in underserved areas, although the efforts were poorly implemented and had very limited effect in decreasing health worker shortages. For example, in 2002, a specific policy was introduced to provide in-service training to physicians working in underserved areas; however, some of the recipient states were not able to provide competent training services and physicians did not abide by the contracts.

To ensure equitable access to quality services for the entire population, there is a need to eliminate health workforce inefficiencies and waste of resources, by improving the productivity and performance of health workers [1, 5]. However, Cameroon is the only one of the four countries that has implemented effective strategies to improve the productivity and performance of health workers; for example, a productivity allowance was introduced, comprising 10% of the financial resources generated by the health facility, to motivate staff. As a result, the quality of the health services increased.

#### Regulation of the private sector

Policies to regulate both the public and private sectors have mainly focused on boosting private sector participation in training health workers. These policies have contributed to the growth in the number of graduates in the four countries over the past decade. However, the lack of government regulation may compromise the quality of training and service delivery. The growth of the private health labour market means that there is a need to develop specific policies to regulate the private sector, to improve the quality of training and service delivery, and to manage dual practice in order to ensure equitable access to quality health services for the entire population.

## Conclusions

An understanding of the interactions between the factors that determine demand and supply of the health workforce – the health labour market dynamics – is critical if countries are to develop effective polices to address health workforce shortages and maldistribution and attain universal health coverage. Partial health workforce policies, such as those that focus just on training more health workers, are not sufficient to reduce health worker shortages.

We found that over the past decade, there have been great improvements in the availability of health workers in Cameroon, Kenya, Sudan, and Zambia. However, despite these improvements, there are persistent health workforce shortages and maldistribution. Several factors contribute to and aggravate the shortage of health workers in the four countries, including migration, an aging workforce, and imbalances in the skill mix composition. The lack of health workforce capacity to provide health services to the entire population is a major challenge for ensuring equitable access to health services and for attaining universal health coverage.

In Kenya and Sudan, increasing the production of health workers, particularly physicians, may not be the most appropriate strategy in the short term to reduce health worker shortages, as these countries experience high vacancy and physician migration rates. In Cameroon and Zambia, however, training of new graduates, coupled with recruitment strategies, such as improving working conditions, safety, and wages to attract health workers back to the health sector, could decrease the geographical inequalities and economic shortage of health workers.

For further policies to be effective in reducing health workforce shortages, the countries will need to take into consideration the dynamics of the health labour market. In general, policies should be directed to recruiting and retaining health workers in underserved areas, and should include strategies to improve the retention, productivity, and quality of the current health workforce. In addition, appropriate regulation of the private sector should be implemented, including monitoring of dual practice. Eliminating health workforce inefficiencies by improving productivity and performance will be critical in increasing the supply of services, and, therefore, to some extent mitigating the shortage of health workers [1]. Measures to improve productivity, such as having the optimal workforce mix and providing the appropriate technology and capital, can be important overall strategies. Finally, changes should be made to the skill mix of health workers, by growing and appropriately training and supervising non-professional cadres of health workers, such as community health workers and other health care providers, to perform a variety of health care tasks [24–26].

## Endnotes

^a^For example, the 2010 UK Coalition Government engaged in retrenchment and restructuring of the public sector [8].

^b^Sudan in this paper refers to former Sudan, before South Sudan gained independence in 2011.

^c^The country analyses were based on a protocol written by R Scheffler in consultation with WHO, aimed at understanding the health labour dynamics and productivity in low- and middle-income countries [18].

^d^In Zambia, health workers receive a recruitment and retention allowance of 25% of their basic monthly salary; those in rural areas also receive a 20–25% rural and remote hardship allowance. In addition, health workers serving in rural areas are entitled to top-up allowances under the health worker retention scheme of 30–70% of their basic monthly salary [21].

^e^Exits are due to attrition, resignation, or internal or external migration.

^f^The analysis of “positive” and “negative” effect is a broad categorization that applies only to the countries in this sample. It is not generalizable to other situations or other countries.
